# Gene Silencing of ANGPTL3 Induces PCSK9: Exploring the Biological Significance in the Hepatoma Huh7 Cell Line

**DOI:** 10.3390/cells15131195

**Published:** 2026-06-30

**Authors:** Ilaria Rossi, Ruolan Chen, Enidia Hazizaj, Maria Giovanna Lupo, Giorgia Marodin, Stijn Cos, Alessandra Giannella, Giulio Ceolotto, Nicola Ferri

**Affiliations:** 1 Department of Pharmaceutical and Pharmacological Sciences, University of Padova, 35131 Padova, Italygiorgia.marodin@phd.unipd.it (G.M.); 2 Department of Medicine, University of Padova, 35128 Padova, Italy; ruolan.chen@phd.unipd.it (R.C.); mariagiovanna.lupo@unipd.it (M.G.L.); alessandra.giannella@unipd.it (A.G.); giulio.ceolotto@unipd.it (G.C.); 3 AB ANALITICA Srl, 35127 Padova, Italy

**Keywords:** ANGPTL3, PCSK9, Hepatocytes, RNA-seq, α1-antitrypsin, LMAN1, FV, FVIII

## Abstract

**Highlights:**

1. RNA-seq analysis identifies differentially regulated genes after ANGPTL3 and/or PCSK9 silencing in Huh7 hepatoma cells.
2. Gene silencing of ANGPTL3 is associated with reduced SERPINA1 and LMAN1 expression in Huh7 cells.
3. ANGPTL3 silencing is associated with increased FV and FVIII mRNA expression and extracellular levels in Huh7 cells.

**Abstract:**

Background: Angiopoietin-like 3 (ANGPTL3) and proprotein convertase subtilisin/kexin type 9 (PCSK9) are key regulators of lipid homeostasis. We have previously shown that gene silencing of ANGPTL3 significantly induces PCSK9 expression in the human hepatoma cell line Huh7. Here, we investigated the biological significance of this regulation in the cultured human hepatoma cell line Huh7. Methods: We performed an RNA-seq analysis in Huh7 cells transfected with siRNA-ANGPTL3, siRNA-PCSK9, and double siRNA-ANGPTL3/PCSK9. Selected findings were assessed by RT-qPCR, Western blotting, and ELISA. Results: Among 13,945 detected transcripts, 192 genes were differentially expressed after ANGPTL3 silencing, 88 after PCSK9 silencing, and 219 after combined ANGPTL3/PCSK9 silencing, compared with scramble-siRNA controls. When ANGPTL3 gene expression was silenced, we observed a compensatory induction in PCSK9 mRNA and protein expression. Bioinformatic analysis revealed that gene silencing of ANGPTL3 or both ANGPTL3/PCSK9 suppresses serpin family A member 1 (SERPINA1), which encodes α1-antitrypsin, and lectin mannose-binding 1 (LMAN1). These data were confirmed by Western blot and RT-PCR analysis. In addition, ANGPTL3-siRNA, alone or combined with PCSK9-siRNA, significantly increased FV and FVIII mRNA expression and secretion in conditioned medium. Conclusions: Our data identified SERPINA1 and LMAN1 as genes downregulated in response to ANGPTL3 silencing in Huh7 hepatoma cells, which was also associated with increased expression of FV and FVIII. Our study suggests a potential link between ANGPTL3 silencing and coagulation-related processes, extending the biological relevance of ANGPTL3 beyond lipid metabolism.

## 1. Introduction

Triglyceride (TG)-rich lipoprotein homeostasis is regulated by different enzymes, physiological inhibitors, and regulators. Among them, angiopoietin-like 3 (ANGPTL3) has gained considerable attention as a pharmacological target for the treatment of dyslipidemia. ANGPTL3 is predominantly produced by the liver, and its circulating levels are relatively stable during feeding states [1]. ANGPTL3 alone, or more potently in complex with ANGPTL8, inhibits lipoprotein lipase (LPL) [2]. LPL inhibition limits the hydrolysis of triglycerides (TGs) in triglyceride-rich lipoproteins (TRLs), including chylomicrons and very-low-density lipoprotein (VLDL) particles, thereby reducing fatty acid release for storage in adipose tissue and energy utilization by oxidative tissues, such as the heart and skeletal muscle [3]. ANGPTL3 is also known to decrease the activity of endothelial lipase (EL) involved in the hydrolysis of phospholipids in high-density lipoprotein (HDL) [4,5]. Thus, ANGPTL3 plays a pivotal role in TG metabolism as an inhibitor of both LPL and EL.

Consistent with its physiological function, individuals with homozygous or compound heterozygous mutations in the *ANGPTL3* gene have lower plasma concentrations of ANGPTL3, TGs, low-density lipoprotein cholesterol (LDL-C) and HDL cholesterol, and a lower risk of atherosclerotic cardiovascular disease (ASCVD) than heterozygous carriers [6,7]. Considering its relevant role in lipid homeostasis, therapeutic inhibitors of ANGPTL3 have been developed to reduce residual ASCVD risk in dyslipidemic patients [8]. For instance, evinacumab, a monoclonal antibody (mAb) targeting circulating ANGPTL3, significantly reduced LDL-C and TG levels by 49% and 50%, respectively, in patients with homozygous familial hypercholesterolemia (HoFH) [9,10]. Conversely, treatment with vupanorsen, a specific antisense oligonucleotide against ANGPTL3, was associated with hepatic steatosis in clinical studies [11]. Furthermore, carriers of compound heterozygous ANGPTL3 loss-of-function alleles exhibited reduced hepatic VLDL–apolipoprotein B (apoB) production, suggesting impaired TG export from the liver [12]. Nonetheless, these variants were not associated with hepatic steatosis [13].

A second key player in lipid homeostasis is PCSK9. Circulating PCSK9 concentrations correlate with plasma LDL-C and TG levels and, importantly, with hepatic TG content measured by proton magnetic resonance spectroscopy [14,15]. In addition, circulating PCSK9 levels were higher in patients with hepatic fat accumulation and correlated with the severity of steatosis [16]. This evidence suggests that both ANGPTL3 and PCSK9 may be implicated in liver fat accumulation through similar or different intracellular pathways. We previously showed that gene silencing of ANGPTL3 induces lipid accumulation in the human hepatoma cell line Huh7 and increases the expression of PCSK9 [17], indicating a possible association between ANGPTL3 downregulation and PCSK9 regulation. In addition, altered circulating levels of PCSK9 have been observed in patients carrying homozygous ANGPTL3 loss-of-function variants [18]. Therefore, in the present study, we used RNA-seq in Huh7 cells following acute ANGPTL3 silencing to characterize the associated transcriptional changes and to explore the potential biological relevance of increased PCSK9 expression. These in vitro findings are intended to provide mechanistic hypotheses that warrant further investigation in more physiologically relevant models and clinical settings.

## 2. Materials and Methods

### 2.1. Cell Culture

Cell culture reagents and plastic supplies were purchased from EuroClone (Milan, Italy) if not otherwise specified. The Huh-7 cell line was maintained in Modified Eagle’s Medium (MEM) supplemented with 10% Fetal Bovine Serum (FBS), 1% penicillin/streptomycin solution (10,000 U/mL and 10 mg/mL, respectively), 1% L-glutamine 200 mM, and 1% non-essential amino acids 100× solution [17].

### 2.2. ANGPTL3 and PCSK9 Silencing

**Figure 1:**
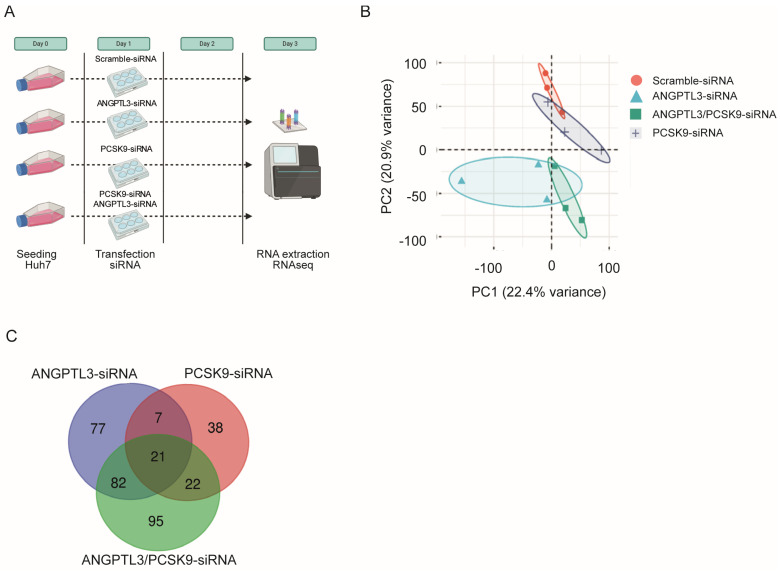
RNA-seq analysis after gene silencing of ANGPTL3 and PCSK9. (**A**) schematic representation of the experimental protocol for RNA-seq analysis. (**B**) Principal component analysis (PCA) of samples selected for RNA-seq data analysis and differential expression genes exploration. (**C**) Venn diagram showing the distribution of differentially expressed transcripts across different conditions. In (panel **B**), colors and symbols identify the treatment groups as indicated in the legend; in (panel **C**), each colored circle represents the indicated siRNA condition.

Gene silencing was performed as previously described [17]. Briefly, cells were seeded and then grown to 70% confluence in MEM/10% FBS. Cells were washed with phosphate-buffered saline (PBS) (SIGMA-Aldrich, St. Louis, MO, USA) and fresh culture medium was added to each plate and then transfected with ANGPTL3-siRNA (Silencer^®^ Pre-designed human ANGPTL3-siRNA: Cat#AM16708; s26160; from Thermo Fisher Scientific, Waltham, MA, USA), or PCSK9-siRNA (siGENOME Human PCSK9-siRNA SMARTpool; Carlo Erba Reagents, Cornaredo, Italy) or both sRNAs together, or scramble-siRNA purchased from Thermo Fisher Scientific. The siRNAs were mixed to obtain a 5 μM stock solution in nuclease-free water. Silencing was performed with 20 nM of siRNA transfected with Lipofectamine™ 3000 Transfection Reagent (Thermo Fisher Scientific, Waltham, MA, USA, Catalog number: L3000001) according to the manufacturer’s instructions. Cells were incubated for 48 h and then processed for the analysis (Figure 1A). To detect silencing efficiency, RT-qPCR and Western blot analysis were performed according to the methods described in Section 2.4 and Section 2.5.

### 2.3. Next-Generation Sequencing

#### 2.3.1. Sample Preparation

On the third day, 48 h after the silencing, the Huh7 cells were washed twice with PBS. The total RNA was extracted using a miRNeasy Micro Kit (Qiagen, Hilden, Germany, ID: 217084). The procedure provided by the manufacturer was followed. The concentration and quality of RNA were measured by NanoDrop^TM^ (Thermo Fisher Scientific, Waltham, MA, USA; ND2000) and Qubit^TM^ 4 Fluorometer (Thermo Fisher Scientific, Waltham, MA, USA; Q33238), both performed according to the manufacturer’s instructions.

#### 2.3.2. RNA-seq Library Preparation and Sequencing

RNA-seq was performed on Huh-7 cells treated with ANGPTL3-siRNA, PCSK9-siRNA, a combination of ANGPTL3 and PCSK9-siRNAs, and scramble siRNA as a control. Each condition was analyzed in triplicate and 200 ng of RNA was used for RNA-seq library preparation using the Illumina Stranded mRNA Prep (Illumina, San Diego, CA, USA). Briefly, purified mRNA was fragmented using oligo (dT) magnetic beads to capture mRNAs with polyA tails. mRNAs were copied into first-strand complementary DNA (cDNA) and second-strand cDNA synthesis. Then, adenine/thymine and ligate adapters were added at the fragment ends. The resulting products were amplified to add indices and primer sequences for cluster generation. A pool of libraries (1.2 pM) mixed with 2% PhiX was sequenced with a High-Output flow cell (75 × 2 cycles, up to 400 M of reads) using NextSeq 550 (Illumina). The number of reads per sample ranged from approximately 30 to 40 million. RNA quality metrics such as RIN values (>9), mapping rates (GRCh38), and the raw sequencing data have been deposited in GEO (GEO Accession GSE312289).

#### 2.3.3. Bioinformatic Analysis on RNA-seq Data

Data analysis was performed using CLC Genomics Workbench software v.20 (Qiagen). Reads from FASTQ files were filtered for quality and aligned to the Ensembl-v99-hg38 version of the reference human genome. Data were normalized using the Trimmed Mean of M-values method. Statistically significant differentially expressed transcripts were identified using Empirical analysis of Differential Gene Expression (EDGE, a count-based statistic) with expression values. An adjusted *p*-value using the false discovery rate (FDR) was considered significant at FDR < 0.05. Principal component analysis (PCA) was visualized in a two-dimensional plot to investigate the clustering of data.

A volcano plot was generated using a fold change of >1.5 and an adjusted *p*-value (FDR) < 0.05. Gene ontology and pathway enrichment analysis of significant differentially expressed genes were conducted by Gene Ontology enrichment analysis (https://geneontology.org (accessed on 30 March 2026)).

### 2.4. Western Blotting

Western blot analysis was performed as previously described [19]. In brief, a total of 200,000 cells/well were seeded and treated 24 h later according to the experiment. After 48 h, cells were washed twice with PBS (SIGMA-Aldrich) and homogenized in lysis buffer containing 1% NP-40, 150 mM NaCl, and 50 mM Tris-HCl at pH 7.5. Protein concentration was assessed by BCA assays (Euroclone), according to the manufacturer’s instructions. The 25-µg total protein extract/samples were separated on 4–20% SDS-PAGE gel (Bio-Rad, San Francisco, Hercules, CA, USA) under denaturing and reducing conditions. Proteins were then transferred onto a nitrocellulose membrane by using the Trans-Blot^®^ Turbo™ Transfer System (Bio-Rad); 5% non-fat dried milk in Tris-buffered saline containing 0.2% of Tween 20 (TBST20) was used as blocking buffer. All the primary antibodies were diluted in 5% non-fat dried milk in TBST20 and incubated overnight at 4 °C in agitation. Horseradish peroxidase (HRP) conjugated secondary antibodies were diluted in blocking solution and membranes were left to incubate for 90 min at room temperature in agitation. Luminescence signals were acquired with Uvitec Alliance Q9 (Uvitec, Cambridge, UK). Quantitative densitometric analysis was performed with ImageJ software v1.54d. When used, the stripping buffer was prepared according to Abcam’s recipe. The PCSK9 antibody was from GeneTex (Irvine, CA, USA) (cod. GTX129859; dilution 1:1000), ANGPTL3 antibody was from GeneTex (cod GTX104569; dilution 1:500), LMAN1 antibody was from GeneTex (cod GTX114530; dilution 1:1000), GAPDH antibody was from GeneTex (cod. GTX100118; dilution 1:5000), and anti-rabbit secondary antibody was from Jackson ImmunoResearch (Cambridge, UK) (cod. 113-036-045, dilution 1:5000).

### 2.5. ELISA Assay for ApoB, FV and FVIII

Cells were seeded in 6-well plates (200,000 cells/well), and treatments were performed 24 h later. After 48 h, conditioned medium was collected, centrifuged at 15,000 rpm for 10 min, and analyzed by ELISA according to the manufacturer’s instructions. Absorbance at 450 nm was measured with a VICTOR Nivo Multimode Microplate Reader (PerkinElmer, Shelton, CT, USA). The Human ApoB ELISA kit was purchased from FineTest (Wuhan Fine Biotech Co., Ltd., Wuhan, China; cod. EH0620). Human FV and FVIII ELISA kits were purchased from BT LAB Bioassay Technology Laboratory, Shanghai, China (code E4114hu and code E3723Hu, respectively).

### 2.6. Reverse Transcription and Quantitative PCR (RT-qPCR)

RT-qPCR was performed as previously described [17]. In brief, total RNA was extracted using the iScript™ RT-qPCR Sample Prep reagent (Bio-Rad), according to the manufacturer’s instructions. QuantiNova SYBR Green RT-PCR Kit (QIAGEN, Hilden, Germany) was used for qPCR, along with specific primers for ANGPTL3 (FWD 5′-GCCTGTTGGAGACTCAGATGG-3′, REV 5′-TAGCACCTTCTGTGCCTGGG-3′), PCSK9 (FWD 5′-CCTGCGCGTGCTCAACT-3′, REV 5′-GCTGGCTTTTCCGAATAAACTC-3′), LMAN1 (FWD 5′-GGCGTCTATGAGACAACACAGC-3′, REV 5′-GGTGGTAGTTCTGGGCATTTCG-3′), SERPINA1 (FWD 5′-GATCAACGATTACGTGGAGAAGG-3′, REV 5′-CCTAAACGCTTCATCATAGGCA-3′), FV (FWD 5′- GCCAGACCTTGCTGGAAAATGG-3′, REV 5′-CCAACCTCTGTGTTTAGGAGCC- 3′), FVIII (FWD 5′-GCCATTGGGAATGGAGAGTAAAG-3′, REV 5′- CCTGAGTAGTTACTCCTGTGAC-3′), 18S (FWD 5′-CGGCTACCACATCCACGGAA-3′, REV 5′-CCTGAATTGTTATTTTTCGTCACTACC-3′), and HMBS (FWD 5′-GGCAATGCGGGCTGCAA-3′, REV 5′- GGGTACCCACGCGAATCAC-3′).

The analyses were performed with the CFX96 Touch Real-Time PCR Detection System (Bio-Rad) with cycling conditions of 45 °C for 10 min, 95 °C for 5 min, and a repetition of 40 cycles at 95 °C for 5 s followed by 30 s at 60 °C. The data were expressed as Ct values and used for relative quantification of targets with ΔΔCt calculations. The ΔΔCt values were determined by multiplying the ratio value between the efficiency of specific primers and housekeeping 18S. The efficiency was calculated as ((10^(−1/slope)^) − 1) × 100.

### 2.7. Statistical Analysis

Data are expressed as mean ± standard deviation. To compare differences between two conditions, *p* values were determined by Student’s *t*-test using GraphPad^®^ Software v8.2.1 for Windows. Otherwise, differences between treatment groups were evaluated by one-way ANOVA. The probability value of *p* < 0.05 was considered statistically significant. Unless otherwise stated, *p* values were >0.05.

## 3. Results

### 3.1. RNA-seq Analysis of Huh7 Cells Transfected with ANGPTL3-siRNA and PCSK9-siRNA

To elucidate the role of ANGPTL3 associated with the regulation of PCSK9 expression on lipid homeostasis, we performed RNA-seq in Huh7 cells transfected with ANGPTL3-siRNA, PCSK9-siRNA, and the combination of both ANGPTL3-siRNA and PCSK9-siRNA (Figure 1A). Principal component analysis of the RNA-seq data revealed clear separation between mRNA transcript profiles across the different treatment groups (Figure 1B). Out of 13,945 detected transcripts, differential expression analysis revealed that 192 genes were significantly altered in Huh7 cells transfected with ANGPTL3-siRNA compared to scramble-siRNA controls (78 upregulated and 114 downregulated, Tables S1 and S2). In cells transfected with PCSK9-siRNA, 88 genes were differentially expressed (49 upregulated and 39 downregulated, Tables S3 and S4), while dual transfection with both ANGPTL3- and PCSK9-siRNA resulted in 219 differentially expressed genes (83 upregulated and 137 downregulated, Tables S5 and S6).

A Venn diagram shows the distribution of differentially expressed transcripts across treatment conditions (Figure 1C). Overall, 21 transcripts were consistently differentially expressed across all conditions relative to scramble-siRNA cells, suggesting a shared regulatory response to siRNA-mediated gene silencing (Figure 1C). In particular, some of these transcripts are involved in lipid metabolism, such as PAQR4 (Progestin And AdipoQ Receptor Family Member 4), CES1 (Carboxylesterase 1), APOD (Apolipoprotein D), and HSD11B2 (Hydroxysteroid 11-Beta Dehydrogenase 2) (Tables S1–S6).

**Figure 2:**
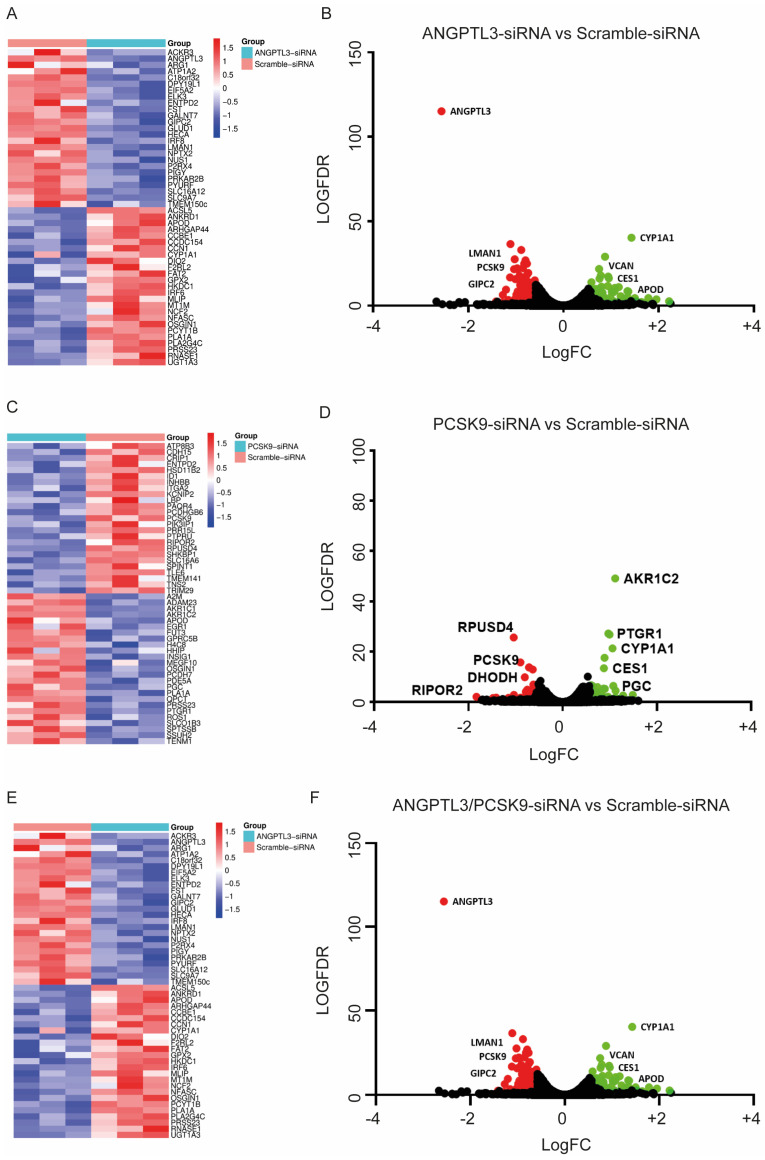
Heat map and volcano plots of differentially expressed genes. (**A**,**C**,**E**) Heat map of upregulated and downregulated mRNA levels in response to ANGPTL3-siRNA (panel **A**), PCSK9-siRNA (panel **C**), and ANGPTL3/PCSK9-siRNA (panel **E**). Data are expressed as the log scale of normalized counts. The heat map represents read-count expression. Different genes are indicated on the right side in the corresponding line. On the top parts of the graphs, the different experimental conditions are indicated. (**B**,**D**,**F**) Volcano plot showing the most relevant differentially expressed genes. Red dots show the downregulated genes and green dots the upregulated genes after transfection with ANGPTL3-siRNA (panel **B**), PCSK9-siRNA (panel **D**), and ANGPTL3/PCSK9-siRNA (panel **F**).

Heat map and volcano plot visualizations illustrate the gene expression profiles across different treatment conditions relative to cells transfected with scramble-siRNA (Figure 2A–F). As expected, the expression levels of ANGPTL3 and PCSK9 mRNA were markedly reduced in cells treated with their respective siRNAs.

### 3.2. Gene-Silencing of ANGPTL3 Leads to PCSK9 Upregulation

**Figure 3:**
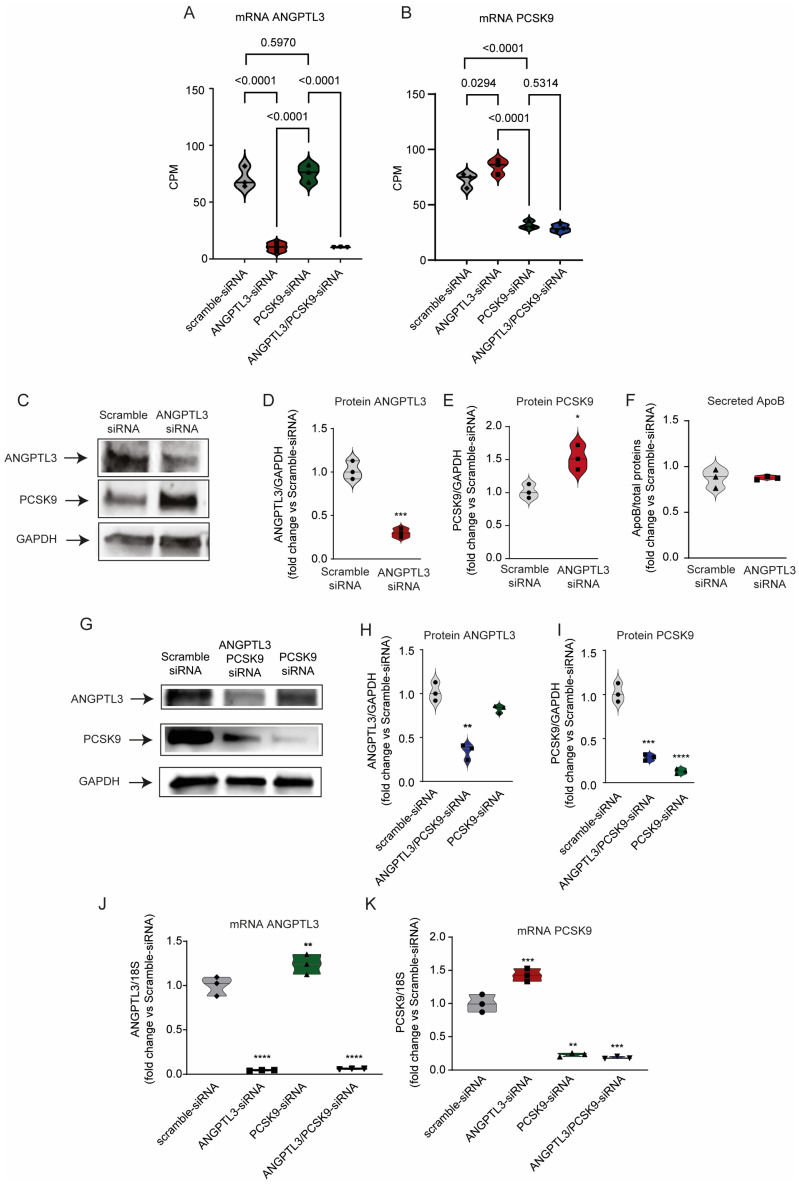
Differential regulation of gene and protein expression of ANGPTL3 and PCSK9 after siRNA transfection. (**A**,**B**) ANGPTL3 and PCSK9 gene expression analysis in response to different siRNA in Huh7 cells. *p* values were calculated using ANOVA one-way Benjamini and Hochberg FDR method. (**C**,**G**) Western blot analysis for ANGPTL3 and PCSK9 after 48 h transfection with scramble-siRNA and ANGPTL3-siRNA (panel **C**) and scramble-siRNA, PCSK9-siRNA, and double siRNA-ANGPTL3/PCSK9 (panel **G**). GAPDH was used as a loading control and quantifications of protein expression are shown in panels (**D**–**F**,**H**,**I**). ApoB secretion was determined by ELISA assay. (**J**,**K**). Real-time quantitative PCR for ANGPTL3 (panel **J**) and PCSK9 (panel **K**) from total RNA extracted from Huh7 cells transfected with indicated siRNA. *p* values were calculated using one-way ANOVA method. * *p* < 0.05; ** *p* < 0.01; *** *p* < 0.001; **** *p* < 0.0001 vs. scramble-siRNA. Data from Western blot and real-time quantitative PCR are presented as mean ± SD of three independent experiments.

In cells transfected with ANGPTL3-siRNA, the expression of PCSK9 transcripts was significantly increased compared to scramble controls (*p* = 0.0294; Figure 3A,B). Therefore, we validated the next generation sequencing (NGS) results from the differential expression analysis by assessing PCSK9 expression in siRNA-transfected cells at both protein and mRNA levels using Western blot and real-time PCR, respectively. Immunoblotting confirmed the increase of PCSK9 protein expression in ANGPTL3-siRNA transfected cells in comparison to scrambled cells (Figure 3C,E). A similar effect was confirmed in HepG2 cells (Figure S1). Gene expression of PCSK9 was significantly enhanced in ANGPTL3-siRNA transfected cells with respect to the scramble cells (Figure 3K). The induction of PCSK9 did not modulate the secretion of ApoB by Huh7 cells, as determined by ELISA assay (Figure 3F).

Starting from the regulation of PCSK9 by siRNA-ANGPTL3, we performed an additional analysis by silencing PCSK9 and both ANGPTL3 and PCSK9. The silencing of PCSK9 was very efficient at both protein and transcript levels (Figure 3G,I,K). The combination of both siRNAs also showed very efficient and almost complete silencing of ANGPTL3 and PCSK9 at protein and mRNA levels (Figure 3G–K). While we observed a partial induction of ANGPTL3 mRNA levels in response to PCSK9-siRNA (Figure 3J), this effect was not evident at the protein level determined by Western blot analysis (Figure 3G,H). Thus, gene silencing of ANGPTL3 was associated with significant upregulation of PCSK9, while the silencing of PCSK9 induced a less pronounced induction of ANGPTL3 at mRNA levels but not at protein levels. As expected, we observed a tendency toward increased expression levels of LDL receptor in response to PCSK9-siRNA and double ANGPTL3/PCSK9 siRNA (Figure S2).

### 3.3. Gene Silencing of ANGPTL3 Leads to Downregulation of SERPINA1 and LMAN1

**Figure 4:**
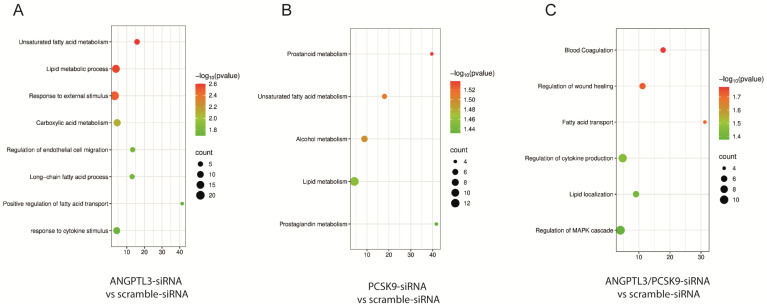
Enrichment analysis by Gene Ontology Biological Processes (GO-BP) database in response to ANGPTL3-siRNA (panel **A**), PCSK9-siRNA (panel **B**), and ANGPTL3/PCSK9-siRNA (panel **C**).

The enrichment analysis was conducted using the Gene Ontology Biological Processes (GO-BP) database to identify biological functions associated with different gene expression profiles under various cellular conditions. In ANGPTL3-siRNA-treated cells, GO-BP analysis revealed significant enrichment of target genes involved in response to external stimuli, unsaturated fatty acid metabolism, positive regulation of fatty acid transport, lipid metabolic processes, and cytokine response (Figure 4A). Specifically, we observed overexpression of genes such as *ACSL5*, *FABP3*, *APOD*, *PLA2G4C*, *APOL1*, *PLA1A*, and *DHCR7*, which are associated with lipid metabolism (Table S2). In PCSK9 siRNA-treated cells, GO-BP analysis showed enrichment in biological processes including alcohol metabolism, prostanoid metabolism, unsaturated fatty acid metabolism, prostaglandin biosynthesis, and general lipid metabolism (Figure 4B). Notably, there was an overexpression of genes belonging to the aldo-keto reductase family (*AKR1C2*) and the cytochrome P450 enzyme family (*CYP1A1*; Table S4). In ANGPTL3/PCSK9-siRNA GO-BP analysis revealed significant enrichment in biological processes such as lipid localization, cytokine production, fatty acid transport, and regulation of blood coagulation (Figure 4C).

**Figure 5:**
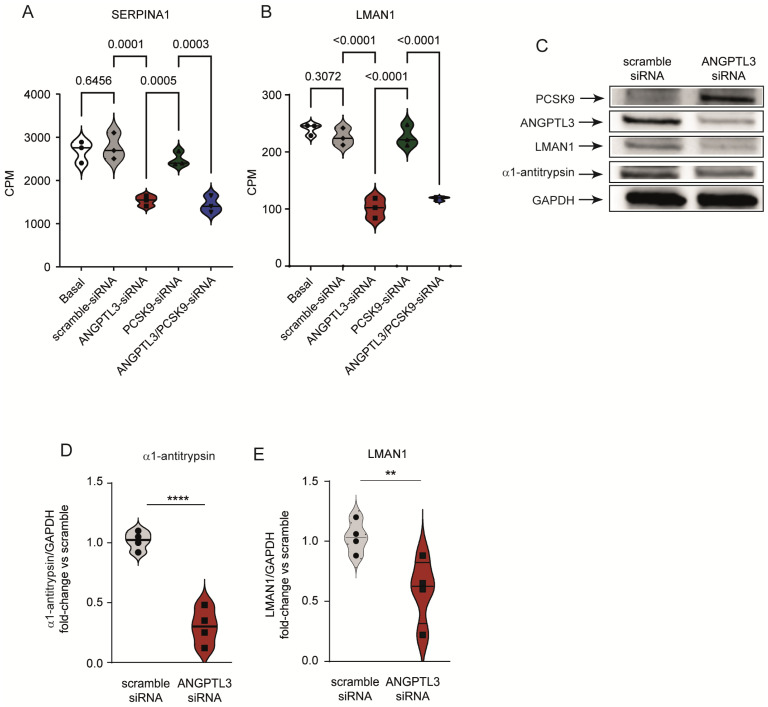
α1-antitrypsin and LMAN1 were downregulated in response to ANGPTL3-siRNA in Huh7 cells. (**A**,**B**) Differential expression of SERPINA1 and LMAN1 determined by NGS after different siRNA transfection. (**C**) Protein analysis from Huh7 cells after transfection with scramble-siRNA and ANGPTL3-siRNA. GAPDH was used as loading control. (**D**,**E**) Quantification analysis of Western blot. *p* values were calculated using ANOVA one-way Benjamini and Hochberg FDR method (**A**,**B**) or Student’s *t*-test (**D**,**E**). ** *p* < 0.01, **** *p* < 0.0001 vs. scramble-siRNA. Western blot analysis is presented as mean ± SD of four independent experiments.

PCSK9-siRNA showed a different pathway regulation compared to ANGPTL3-siRNA, which instead appeared very similar to the ANGPTL3/PCSK9 double gene silencing. SERPINA1 (serpin family A member 1) and LMAN1 (lectin, mannose binding 1) genes and proteins were downregulated in response to ANGPTL3-siRNA and ANGPTL3/PCSK9-siRNA (Tables S1 and S5), as also confirmed by real-time PCR and Western blot analysis, respectively (Figure 5A–E).

### 3.4. Gene Silencing of ANGPTL3 Is Associated with Increased Levels of Coagulation Factors V and VIII

**Figure 6:**
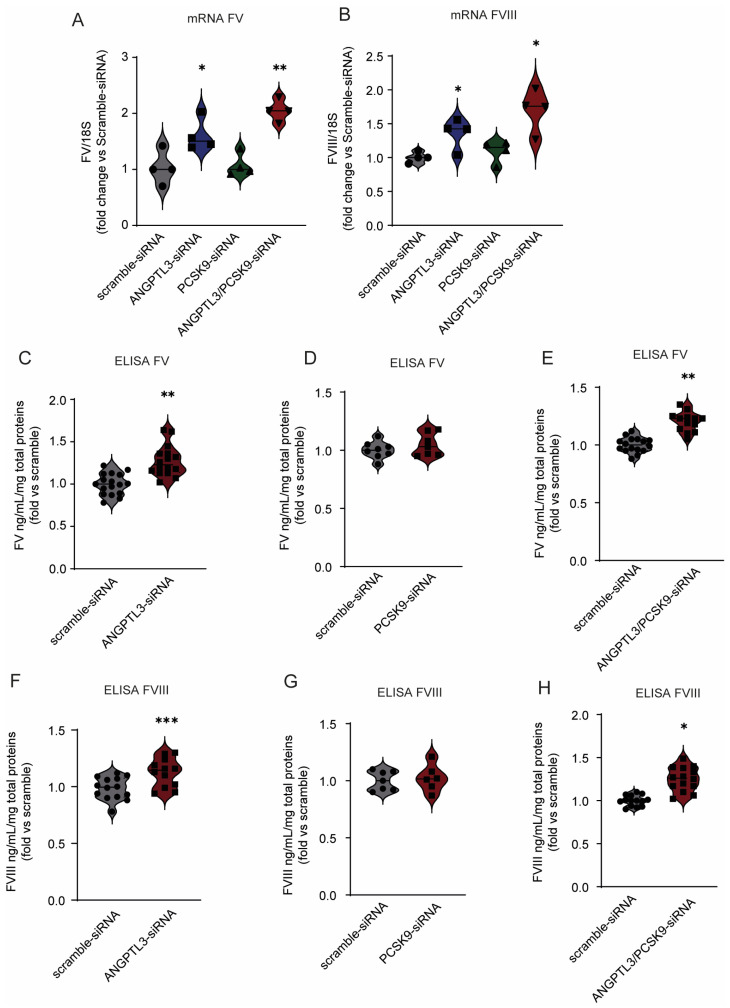
FV and FVIII were significantly upregulated in response to ANGPTL3-siRNA. (**A**,**B**) FV and FVIII mRNA were determined by RT-qPCR after 48 h of different siRNA transfection. *p* values were calculated using one-way ANOVA method. * *p* < 0.05; ** *p* < 0.01; vs. scramble-siRNA. (**C**–**H**) ELISA assay for FV (**C**–**E**) and FVIII (**F**–**H**) from conditioned media of Huh7 cells after 48 h of different siRNA transfection. FV and FVIII determinations were normalized for total protein content of cell lysates. *p* values were calculated using Student’s *t*-test. * *p* < 0.05; ** *p* < 0.01, *** *p* < 0.001 vs. scramble-siRNA.

As shown from the STRING analysis, SERPINA1 is connected to ANGPTL3 through SAR1B (secretion-associated Ras-related GTPase 1B) (Figure S3). More intriguingly, LMAN1, together with MCFD2 (multiple coagulation factor deficiency 2), has been shown to form a cargo receptor complex required for the efficient secretion of FV and FVIII [20,21]. In addition, α1-antitrypsin has been reported as a potential cargo protein dependent on LMAN1 (ERGIC-53) for efficient secretion [22]. Given the significant enrichment of genes involved in the regulation of blood coagulation observed following ANGPTL3/PCSK9 silencing, we investigated the release of FV and FVIII from Huh7 cells. We first investigated the mRNA levels of both FV and FVIII by RT-qPCR in total RNA extracts from Huh7 cells after transfection with ANGPTL3 siRNA, PCSK9 siRNA, or double siRNA. Interestingly, FV and FVIII mRNA levels were induced after gene silencing of ANGPTL3 alone or in combination with PCSK9, whereas PCSK9 silencing alone did not produce significant effects (Figure 6A,B). The increase in FV mRNA levels was observed after normalization to both 18S and HMBS (hydroxymethylbilane synthase) housekeeping genes (Figure 6A and Figure S4). To further support these findings, we measured extracellular levels of FV and FVIII in conditioned media by ELISA assay. Both coagulation factors were significantly upregulated in response to ANGPTL3-siRNA or ANGPTL3/PCSK9-siRNA. Specifically, FV increased by 24% following ANGPTL3 silencing and by 21% following double silencing, whereas FVIII increased by 11% and 21%, respectively (Figure 6C–H). Similar results were observed in the HepG2 cell line with a significant increase in FV secretion in response to ANGPTL3-siRNA and ANGPTL3/PCSK9-siRNA determined by ELISA (Figure S5). However, FVIII secretion and the expression of SERPINA1 and LMAN1 were not evaluated in HepG2 cells. Therefore, these findings should be interpreted as an association observed primarily in Huh7 cells and require confirmation in additional hepatic models.

## 4. Discussion

In the present study, we investigated the biological effects of ANGPTL3 gene silencing in Huh7 hepatoma cells using next-generation sequencing (NGS). RNA-seq analysis showed that ANGPTL3 suppression modulated genes involved in multiple biological pathways, and that most of these changes were retained when PCSK9 was silenced together with ANGPTL3. In contrast, PCSK9 silencing alone induced a more limited transcriptional response. These findings suggest that ANGPTL3 silencing has a predominant role in shaping the transcriptional profile of Huh7 cells, largely independently of PCSK9. However, they should not be interpreted as evidence of a direct regulatory hierarchy between these proteins in vivo.

Among the differentially regulated genes identified following ANGPTL3 silencing, we found a strong downregulation of both SERPINA1 and LMAN1. LMAN1 is an ER-Golgi cargo receptor involved in the transport of several secretory proteins, including FV and FVIII, as well as α1-antitrypsin encoded by SERPINA1 [23,24]. Therefore, the concomitant downregulation of SERPINA1 and LMAN1 identifies a potential association between acute ANGPTL3 silencing and hepatic secretory-pathway genes. Nevertheless, the present data do not demonstrate altered ER-to-Golgi trafficking, reduced secretion efficiency, or a direct mechanistic connection between LMAN1 expression and FV/FVIII release. Future studies could also investigate whether ANGPTL3 silencing affects the release of extracellular vesicles (EVs) containing miRNAs that may modulate lipid homeostasis in extrahepatic tissues. Indeed, very recently, exosomal miR-9-5p has been shown to suppress the expression of HMG-CoA reductase and cholesterol 25-hydroxylase, two enzymes involved in cholesterol synthesis [25].

A relevant and unexpected finding of our study was the enrichment of blood-coagulation-related genes, particularly after combined ANGPTL3/PCSK9 silencing. Based on the role of LMAN1 in FV and FVIII transport [21,24], we further assessed the expression and extracellular concentrations of these coagulation factors. RT-qPCR analysis showed that ANGPTL3 silencing increased FV and FVIII mRNA expression and was associated with modest increases in their concentrations in conditioned medium (+11% to +24% versus scramble-siRNA). A similar pattern was observed after combined ANGPTL3/PCSK9 silencing, whereas PCSK9 silencing alone had no relevant effect. However, our analyses do not explain whether or how LMAN1 downregulation contributed to the observed increase in FV and FVIII release. This finding is counterintuitive because mutations in LMAN1, also known as ERGIC-53, result in combined deficiency of FV and FVIII, an autosomal recessive bleeding disorder characterized by coordinated reductions in both clotting proteins [20]. Therefore, the observed association between reduced LMAN1 expression and increased extracellular FV/FVIII concentrations should not be interpreted as evidence of a direct mechanistic relationship. We also recognize that our study was conducted primarily in hepatoma cell lines and that these findings should be validated in primary human hepatocytes and other physiologically relevant models. In addition, only one pre-designed ANGPTL3 siRNA sequence was used, and no rescue experiment was performed; therefore, potential off-target effects of siRNA-mediated ANGPTL3 silencing cannot be fully excluded.

From this perspective, it is relevant to note that patients with α1-antitrypsin deficiency exhibit a lower prevalence of cardiovascular disease [26,27] and lower levels of remnant cholesterol and TGs, while no differences have been observed in total cholesterol, HDL, or LDL levels [28]. Thus, it is tempting to speculate that α1-antitrypsin deficiency could partially interfere with chylomicron and VLDL secretion by the gastrointestinal tract and liver, respectively. Nevertheless, the association between α1-antitrypsin deficiency and cardiovascular risk is very complex and remains controversial, potentially depending on several factors, such as pre-existing disease and oxidative stress [29].

More intriguingly, in a small cohort of patients with emphysema receiving therapy with α1-antitrypsin (Prolastin), the plasma levels of ANGPTL4 were higher than in untreated matched controls [30]. This effect seems to depend on the amount of fatty acids bound to α1-antitrypsin [31].

Consistent with liver transcriptome profiles of wild-type vs. Serpina1-null mice [32], we found a similar pattern of gene regulation with significant changes in metallothionein-1 and solute carrier family members (*SLC16A12*; *SLC38A11*; *SLC38A6*; *SLC5A3*; *SLC9A7*). In addition, Serpina1-null mice also showed significant changes in ANGPTL3 expression, further supporting a potential association between these pathways [32].

We also confirmed that ANGPTL3 silencing was associated with increased PCSK9 expression, while PCSK9 silencing increased ANGPTL3 expression to a lesser extent [17]. The clinical relevance of these in vitro observations remains unknown; our previous work showed the regulation of PCSK9 levels after inhibition of ANGPTL3. However, these data should not be directly extrapolated to clinical ANGPTL3-targeting therapies. Indeed, in patients with HoFH treated with the anti-ANGPTL3 mAb, evinacumab, increased plasma levels of ANGPTL3 were associated with a tendency toward reduced PCSK9 levels [10]. Importantly, the transcriptional profile following combined ANGPTL3/PCSK9 silencing closely resembled that observed following ANGPTL3 silencing alone, supporting the view that PCSK9 upregulation is unlikely to be the main driver of the lipid and secretory changes observed after ANGPTL3 silencing in Huh7 cells.

Taken together, our data indicate that ANGPTL3 silencing in hepatoma cells is associated with changes in coagulation-related gene expression and extracellular FV/FVIII concentrations, extending the biological relevance of ANGPTL3 beyond lipid metabolism. These observations are exploratory and warrant further investigation in more physiologically relevant models.

## 5. Conclusions

In conclusion, this study identifies ANGPTL3 silencing as a condition associated with coordinated transcriptional and secretory changes in Huh7 cells, including altered FV and FVIII expression and release. The RNA-seq data further indicate that the response is predominantly driven by ANGPTL3 silencing rather than by PCSK9 modulation. Although LMAN1 and SERPINA1 emerged as relevant transcriptional changes, their contribution to the observed phenotype remains to be clarified. These findings provide a basis for future studies addressing the hepatic consequences of intracellular ANGPTL3 modulation.

## Data Availability

The data presented in this study are available on request from the corresponding author.

## Supplementary Materials

The following supporting information can be downloaded at https://www.mdpi.com/article/10.3390/cells15131195/s1, Figure S1: Western blot analysis showing the induction of PCSK9 in HepG2 cells transfected with ANGPTL3-siRNA; Figure S2: Western blot analysis showing a higher LDL receptor expression in Huh7 cells transfected with PCSK9-siRNA; Figure S3: STRING analysis showing the directly connected genes both from textmining and curated databases. SERPINA1 connects through SAR1B to ANGPTL3; Figure S4: RT-qPCR analysis for FV normalized with HMBS housekeeping gene; Figure S5: ELISA assay showing a significant increase in FV secretion in response to ANGPTL3-siRNA in HepG2 cells. Table S1: mRNA downregulated in ANGPTL3-siRNA vs. scramble-siRNA; Table S2: mRNA upregulated in ANGPTL3-siRNA vs. scramble-siRNA; Table S3: mRNA downregulated in PCSK9-siRNA vs. scramble-siRNA; Table S4: mRNA upregulated in PCSK9-siRNA vs. scramble-siRNA; Table S5: mRNA downregulated in ANGPTL3/PCSK9-siRNA vs. scramble-siRNA; Table S6: mRNA upregulated in ANGPTL3/PCSK9-siRNA vs. scramble-siRNA.

## Abbreviations

The following abbreviations are used in this manuscript:

ANGPTL3	Angiopoietin-like 3
ApoB	Apolipoprotein B
APOD	Apolipoprotein D
ASCVD	Atherosclerotic cardiovascular disease
cDNA	Complementary DNA
CES1	Carboxylesterase 1
EDGE	Empirical analysis of Differential Gene Expression
EL	Endothelial lipase
FDR	False discovery rate
FBS	Fetal Bovine Serum
FV	Factor V
FVIII	Factor VIII
GO-BP	Gene Ontology Biological Processes
HDL HMBS	High-density lipoprotein Hydroxymethylbilane synthase
HoFH	Homozygous familial hypercholesterolemia
HRP	Horseradish peroxidase
HSD11B2	Hydroxysteroid 11-Beta Dehydrogenase 2
LDL	Low-density lipoprotein
LDL-C	Low-density lipoprotein-cholesterol
LMAN1	Lectin, mannose-binding 1
LPL	Lipoprotein lipase
mAb	Monoclonal antibody
MCFD2	Multiple Coagulation Factor Deficiency 2
MEM	Modified Eagle’s Medium
NGS	Next-generation sequencing
PAQR4	Progestin And AdipoQ Receptor Family Member 4
PBS	Phosphate-buffered saline
PCA	Principal component analysis
PCSK9	Proprotein convertase subtilisin/kexin type 9
SAR1B	Secretion-associated Ras-related GTPase 1B
siRNA	Small interfering RNAs
SERPINA1	Serpin family A member 1
TGs	Triglycerides
TRLs	Triglyceride-rich lipoproteins
VLDL	Very-low-density lipoprotein
