# Supplementary material for: Gene Silencing of ANGPTL3 Induces PCSK9: Exploring the Biological Significance in the Hepatoma Huh7 Cell Line
Source: Cells. 2026 Jun 30;15(13):1195. doi: 10.3390/cells15131195 (PMC13360153; doi:10.3390/cells15131195)
Supplement: Supplementary file 1 [file cells-15-01195-s001.zip › cells-4314747-supplementary.pdf]

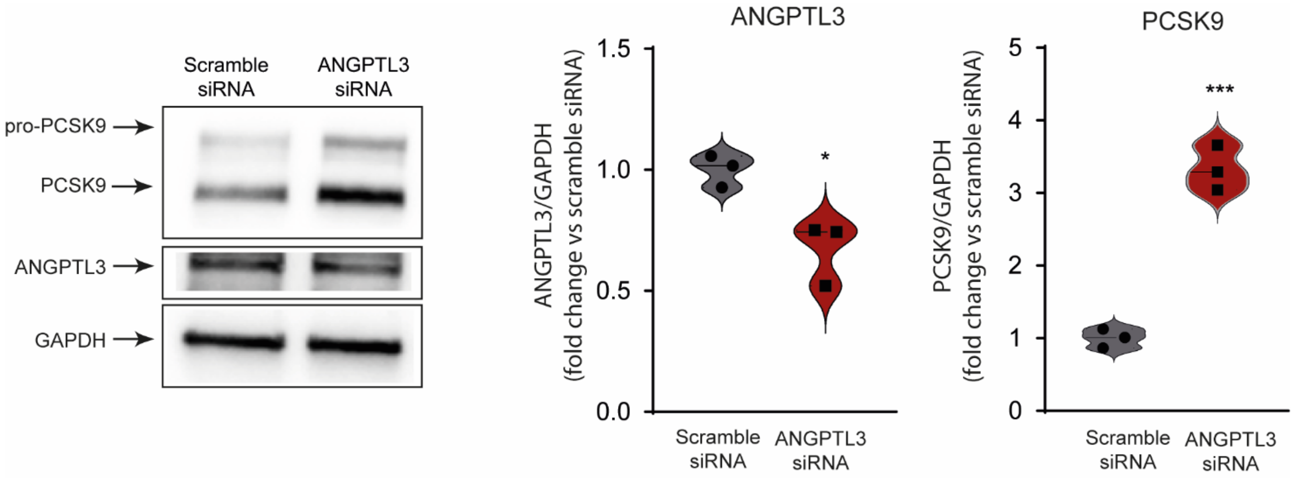

Figure S1. PCSK9 is induced in HepG2 cells transfected with ANGPTL3-siRNA. Western blot analysis for ANGPTL3 and PCSK9 after 48h transfection with scramble-siRNA and ANGPTL3-siRNA. GAPDH was used as loading control and quantification of protein expression are shown in right panels. P values were calculated using Student's t-test method. *p<0.05; ***p<0.001 vs scramble-siRNA. Data are presented as mean ± SD of three independent experiments.

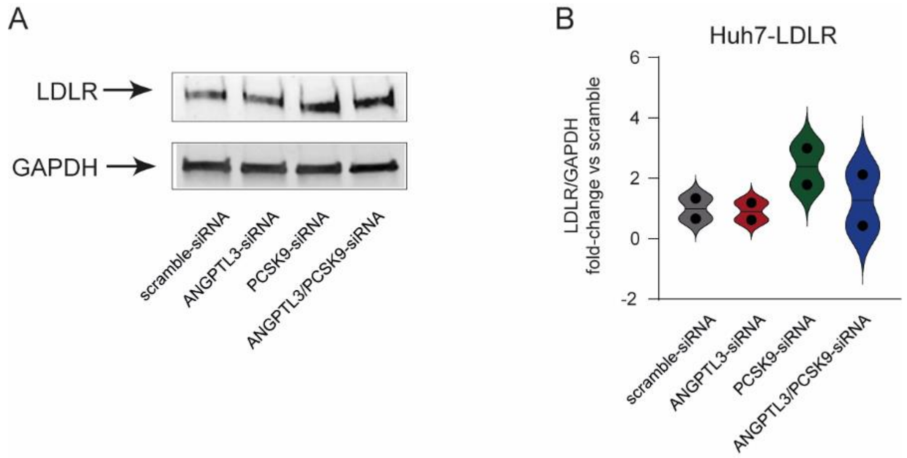

Figure S2. PCSK9-siRNA induces LDL receptor expression in Huh7 cells. A) Western blot analysis for LDL receptor (LDLR) after 48h transfection with scramble-siRNA, ANGPTL3-siRNA, and double siRNA-ANGPTL3/PCSK9. B) GAPDH was used as loading control and quantification of protein expression are shown in panel B.

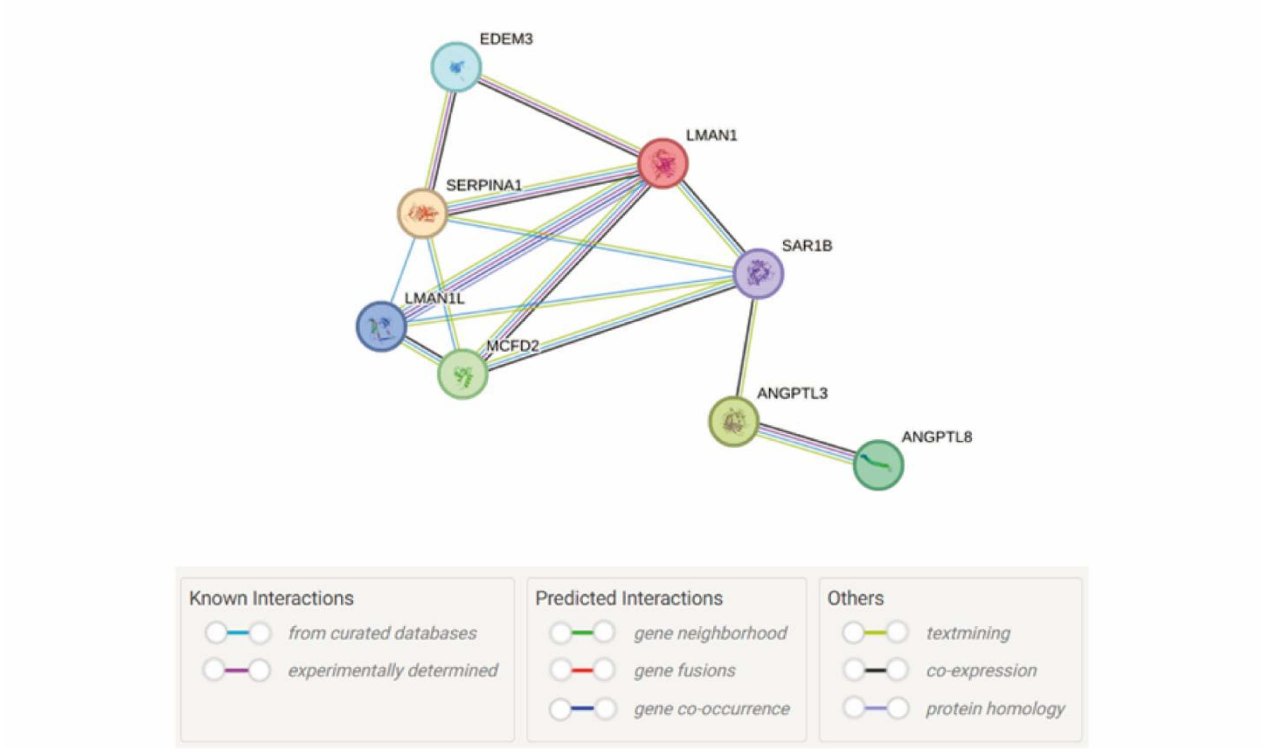

Figure S3. STRING analysis of ANGPTL3, SERPINA and LAMN1.

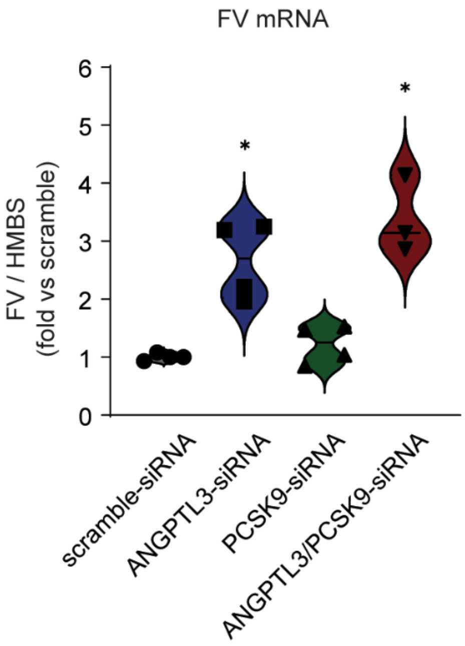

Figure S4. FV was significantly upregulated in response to ANGPTL3-siRNA. FV mRNA were determined by RT-qPCR after 48h of different siRNA transfection and normalized with HMBS housekeeping gene.

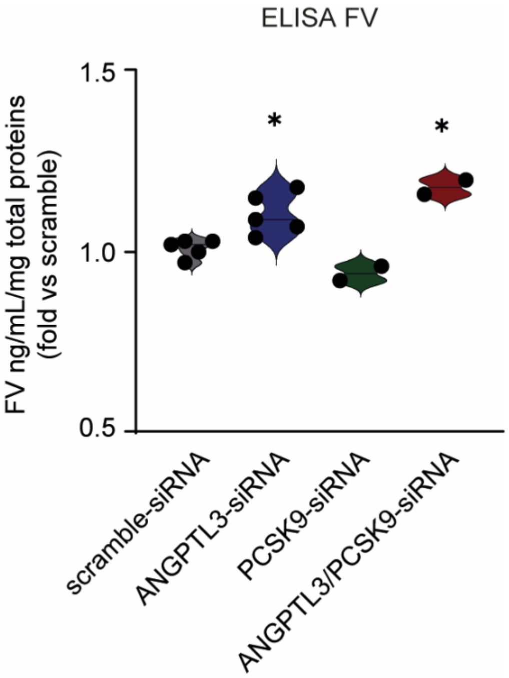

Figure S5. FV was significantly upregulated in response to ANGPTL3-siRNA in HepG2 cells. A) ELISA assay for FV from conditioned media of HepG2 cells after 48h of different siRNA transfection. FV determination was normalized for total protein content of cell lysates. P values were calculated using Student's T-test. *p<0.05 vs scramble-siRNA.

Table S1. mRNA downregulated in ANGPTL3-siRNA vs scramble-siRNA.

Symbol	Gene Name	Log (FC)	Log10 (P adjusted)
ACKR3	Atypical Chemokine Receptor 3	-1.107	1.91
AIFM3	AIF Family Member 3	-0.855	1.8
ALG10	ALG10 Alpha-1,2-Glucosyltransferase	-0.63	2.19
ANGPTL3	Angiopoietin Like 3	-2.520	71.9
ARG1	Arginase 1	-0.646	3.56
ATP1A2	ATPase Na+/K+ Transporting Subunit Alpha 2	-2.024	1.36
ATP6V1G1	ATPase H+ Transporting V1 Subunit G1	-0.731	16.1
B4GALNT4	Beta-1,4-N-Acetyl-Galactosaminyltransferase 4	-1.050	3.86
B4GALT5	Beta-1,4-Galactosyltransferase 5	-1.055	34.1
BGN	Biglycan	-0.593	1.68
C11orf71	Chromosome 11 Open Reading Frame 71	-0.614	1.68
C18orf32	Chromosome 18 Open Reading Frame 32	-0.979	7.58
C2orf15	Chromosome 2 Open Reading Frame 15	-0.752	3.83
C5orf24	Chromosome 5 Open Reading Frame 24	-0.775	13.78
C9orf40	Chromosome 9 Open Reading Frame 40	-0.722	3.85
CBFB	Core-Binding Factor Subunit Beta	-0.699	11.6
CCDC183	Coiled-Coil Domain Containing 183	-1.052	4.62
CCNJ	Cyclin J	-0.798	17.1
CIP2A	Cellular Inhibitor Of PP2A	-0.709	3.66
COCH	Cochlin	-0.613	8.08
COX14	Cytochrome C Oxidase Assembly Factor COX14	-0.612	6.92
CPOX	Coproporphyrinogen Oxidase	-0.606	10.5
CRIP1	Cysteine Rich Protein 1	-0.606	2.75
CRYL1	Crystallin Lambda 1	-0.642	3.04
DCTPP1	DCTP Pyrophosphatase 1	-0.591	6.87
DDAH1	Dimethylarginine Dimethylaminohydrolase 1	-0.768	13.7
DENND6A	DENN Domain Containing 6A	-1.004	19.5
DPY19L1	Dpy-19 Like C-Mannosyltransferase 1	-1.020	29.7
DUSP19	Dual Specificity Phosphatase 19	-0.669	1.4
EIF5A2	Eukaryotic Translation Initiation Factor 5A2	-1.123	12.9
ELK3	ETS Transcription Factor ELK3	-1.139	21.7
ENPP2	Pyrophosphatase/Phosphodiesterase 2	-0.716	21.7
ENTPD2	Ectonucleoside Triphosphate Diphosphohydrolase 2	-1.078	1.6
FBXO32	F-Box Protein 32	-0.707	2.82
FBXO36	F-Box Protein 36	-0.626	2.28
FGD1	FYVE, RhoGEF And PH Domain Containing 1	-0.719	2.79
FKBP14	FKBP Prolyl Isomerase 14	-0.621	6.33
FST	Follistatin	-0.836	14.1
FZD2	Frizzled Class Receptor 2	-0.631	2.35
GALNT7	Polypeptide N-Acetylgalactosaminyltransferase 7	-0.737	12.2
GIPC2	GIPC PDZ Domain Containing Family Member 2	-1.158	9.11
GK5	Glycerol Kinase 5	-0.628	2.6

GLUD1	Glutamate Dehydrogenase 1	-0.928	36.3
GMCL1	Germ Cell-Less 1, Spermatogenesis Associated	-0.712	12.3
GOPC	Golgi Associated PDZ And Coiled-Coil Motif Containing	-0.672	11.08
HAVCR1	Hepatitis A Virus Cellular Receptor 1	-0.79	16
HECA	Hdc Homolog, Cell Cycle Regulator	-1.008	21.7
HNF1B	HNF1 Homeobox B	-0.738	13.6
HSD11B2	Hydroxysteroid 11-Beta Dehydrogenase 2	-0.83	13.5
IL1RAP	Interleukin 1 Receptor Accessory Protein	-0.967	19.5
INPP5A	Inositol Polyphosphate-5-Phosphatase A	-0.632	10.3
IRF8	Interferon Regulatory Factor 8	-0.962	9.24
KLF5	KLF Transcription Factor 5	-0.708	10.4
LEFTY1	Left-Right Determination Factor 1	-0.788	1.5
LHX2	LIM Homeobox 2	-0.826	1.49
LMAN1	Lectin, Mannose Binding 1	-1.237	40.9
LPAR1	Lysophosphatidic Acid Receptor 1	-0.862	4.98
NOTCH2NLB	Notch 2 N-Terminal Like B	-0.905	2.18
NPDC1	Neural Proliferation, Differentiation And Control 1	-0.6	2.08
NPTX2	Neuronal Pentraxin 2	-0.849	8.01
NR0B2	Nuclear Receptor Subfamily 0 Group B Member 2	-0.596	4.49
NRXN3	Neurexin 3	-0.7	2.13
NTS	Neurotensin	-0.666	1.92
NUS1	NUS1 Dehydrodolichyl Diphosphate Synthase	-0.634	13.6
P2RX4	Purinergic Receptor P2X 4	-0.735	11.06
PAFAH1B2	Platelet Activating Factor Acetylhydrolase 1b	-0.784	21
PAN3	Poly(A) Specific Ribonuclease Subunit PAN3	-0.601	7.54
PAQR4	Progestin And AdipoQ Receptor Family Member 4	-0.644	3.49
PCDHGB1	Protocadherin Gamma Subfamily B, 1	-0.607	1.82
PIGY	Phosphatidylinositol Glycan Anchor Biosynthesis Class Y	-1.020	16.5
PKD2	Polycystin 2, Transient Receptor Potential Cation	-0.624	8.64
PLAG1	PLAG1 Zinc Finger	-0.721	6.54
POLA2	DNA Polymerase Alpha 2, Accessory Subunit	-0.61	1.853
POM121	POM121 Transmembrane Nucleoporin	-0.699	20.7
POM121C	POM121 Transmembrane Nucleoporin C	-0.754	20.9
PRKAR2B	Protein Kinase CAMP-Dependent Type II Regulatory Subunit Beta	-1.068	2.58
PTH1R	Parathyroid Hormone 1 Receptor	-1.111	1.38
PYURF	PIGY Upstream Open Reading Frame	-1.004	13.9
RAB2A	RAB2A, Member RAS Oncogene Family	-0.899	21.9
RAB40B	RAB40B, Member RAS Oncogene Family	-0.97	5.73
RARG	Retinoic Acid Receptor Gamma	-0.882	1.58
RNASEH2A	Ribonuclease H2 Subunit A	-0.642	7.11
ROCK1	Rho Associated Coiled-Coil Containing Protein Kinase 1	-0.648	8.88
SEMA6B	Semaphorin 6B	-0.6	7.14
SERPINA1	Serpin Family A Member 1	-0.807	11
SGPP1	Sphingosine-1-Phosphate Phosphatase 1	-0.713	6.85
SLC16A12	Solute Carrier Family 16 Member 12	-1.084	3.04

SLC22A5	Solute Carrier Family 22 Member 5	-0.684	3.92
SLC38A6	Solute Carrier Family 38 Member 6	-0.704	6.61
SLC9A7	Solute Carrier Family 9 Member A7	-0.909	7.59
SMAD9	SMAD Family Member 9	-0.591	3.67
SRGAP2B	SLIT-ROBO Rho GTPase Activating Protein 2B	-0.611	5.06
SYNGR1	Synaptogyrin 1	-0.595	1.82
TBX10	T-Box Transcription Factor 10	-0.744	1.32
TFG	Trafficking From ER To Golgi Regulator	-0.599	15.1
TMEM150C	Transmembrane Protein 150C	-0.663	1.88
TMEM200B	Transmembrane Protein 200B	-0.624	4.78
TMEM33	Transmembrane Protein 33	-0.595	9.81
TMEM64	Transmembrane Protein 64	-1.082	16
TMEM69	Transmembrane Protein 69	-0.687	13
TMEM87B	Transmembrane Protein 87B	-0.651	7.35
TMTC2	Transmembrane O-Mannosyltransferase Targeting Cadherins 2	-0.901	2.2
TNFSF10	TNF Superfamily Member 10	-0.603	7.67
TPRG1L	Tumor Protein P63 Regulated 1 Like	-0.871	17.9
TTC8	Tetratricopeptide Repeat Domain 8	-0.677	3.13
UCK2	Uridine-Cytidine Kinase 2	-0.61	13.3
UHRF1	Ubiquitin Like With PHD And Ring Finger Domains 1	-0.821	6.33
UXS1	UDP-Glucuronate Decarboxylase 1	-0.627	10.1
VNN2	Vanin 2	-0.651	5.02
ZC3H8	Zinc Finger CCCH-Type Containing 8	-0.712	6.04
ZNF100	Zinc Finger Protein 100	-0.848	5.26
ZNF253	Zinc Finger Protein 253	-0.592	8.2
ZNF28	Zinc Finger Protein 28	-0.599	9.6
ZNF681	Zinc Finger Protein 681	-0.758	3.08

Table S2. mRNA upregulated in ANGPTL3-siRNA vs scramble-siRNA.

Symbol	Gene Name	Log (FC)	Log10 (P adjusted)
ACSL5	acyl-CoA synthetase long chain family member 5	0.65	9.19
AKAP12	A-kinase anchoring protein 12	0.70	7.37
ALPK3	alpha kinase 3	0.71	5.38
ANKRD1	ankyrin repeat domain 1	1.24	3.98
ANXA1	annexin A1	0.63	1.62
APOD	apolipoprotein D	1.18	3.73
APOL1	apolipoprotein L1	0.86	3.06
ARHGAP44	Rho GTPase activating protein 44	1.42	2.07
ARL4C	ADP ribosylation factor like GTPase 4C	0.69	1.73
CCBE1	collagen and calcium binding EGF domains 1	1.45	1.49
CCDC154	coiled-coil domain containing 154	1.80	1.57
CCN1	cellular communication network factor 1	0.93	6.98
CD96	CD96 molecule	0.72	2.29
CES1	NA	1.17	26.24
CFAP251	cilia and flagella associated protein 251	1.18	4.42
CNTNAP2	NA	0.83	5.98
CSF1	colony stimulating factor 1	0.59	9.59
CYP1A1	cytochrome P450 family 1 subfamily A member 1	1.35	35.76
CYP2A7	cytochrome P450 family 2 subfamily A member 7	0.62	2.30
CYP7A1	cytochrome P450 family 7 subfamily A member 1	0.88	1.65
DCDC1	doublecortin domain containing 1	0.70	4.56
DDX60	DExD/H-box helicase 60	0.73	3.16
DHCR7	7-dehydrocholesterol reductase	0.61	4.94
DIO2	iodothyronine deiodinase 2	1.50	1.41
DNAH5	dynein axonemal heavy chain 5	0.85	1.67
EDN1	endothelin 1	0.70	2.14
F2RL2	coagulation factor II thrombin receptor like 2	1.30	3.38
FABP3	fatty acid binding protein 3	0.60	4.56
FAT2	FAT atypical cadherin 2	1.08	1.75
G0S2	G0/G1 switch 2	0.92	6.33
GPX2	glutathione peroxidase 2	0.77	5.32
H1-2	H1.2 linker histone, cluster member	0.72	6.39
H2BC5	H2B clustered histone 5	0.63	3.41
H4C8	H4 clustered histone 8	0.79	4.35
HDAC9	histone deacetylase 9	0.74	4.20
HKDC1	hexokinase domain containing 1	1.08	8.56
IFI6	interferon alpha inducible protein 6	0.72	2.48
IGSF23	immunoglobulin superfamily member 23	0.59	3.20
IL15	interleukin 15	0.85	2.67
IQCN	IQ motif containing N	0.79	1.64
IRF6	interferon regulatory factor 6	1.85	5.10
JAML	junction adhesion molecule like	0.70	5.97
LRRC7	leucine rich repeat containing 7	0.59	1.97

MAP3K9	mitogen-activated protein kinase kinase kinase 9	0.60	9.97
MLIP	muscular LMNA interacting protein	1.52	2.76
MT1F	metallothionein 1F	0.81	5.56
MT1M	metallothionein 1M	1.25	1.38
MX1	MX dynamin like GTPase 1	0.86	2.60
MYOF	myoferlin	0.94	4.84
NCF2	neutrophil cytosolic factor 2	1.64	3.96
NFASC	neurofascin	1.86	2.58
NRG1	neuregulin 1	1.38	9.42
NRP2	neuropilin 2	0.72	7.13
OAS1	2'-5'-oligoadenylate synthetase 1	0.81	8.81
OAS3	2'-5'-oligoadenylate synthetase 3	0.75	16.61
OSGIN1	oxidative stress induced growth inhibitor 1	0.77	12.65
PCED1B	PC-esterase domain containing 1B	0.74	6.13
PCYT1B	phosphate cytidylyltransferase 1B, choline	1.03	1.37
PHLDA1	pleckstrin homology like domain family A member 1	0.62	6.45
PLA1A	phospholipase A1 member A	0.96	13.66
PLA2G4C	phospholipase A2 group IVC	0.72	4.74
PLCXD3	phosphatidylinositol specific phospholipase C X domain containing 3	1.16	1.44
PPIP5K1	diphosphoinositol pentakisphosphate kinase 1	0.63	4.56
PRODH	proline dehydrogenase 1	0.63	3.62
PRSS23	serine protease 23	1.05	8.87
PTPRR	protein tyrosine phosphatase receptor type R	1.67	1.57
QPCT	glutaminyl-peptide cyclotransferase	0.67	2.76
RNASE1	ribonuclease A family member 1, pancreatic	2.20	2.67
RUNX1	RUNX family transcription factor 1	0.60	4.44
SLC1A3	solute carrier family 1 member 3	0.71	3.49
SSUH2	ssu-2 homolog	1.67	13.76
SULT1C2	sulfotransferase family 1C member 2	0.65	5.46
SUSD4	sushi domain containing 4	0.61	5.06
TENT5C	terminal nucleotidyltransferase 5C	1.03	4.09
TNFRSF19	TNF receptor superfamily member 19	0.91	16.54
TUBA1A	tubulin alpha 1a	0.78	13.94
UGT1A3	UDP glucuronosyltransferase family 1-member A3	1.77	3.08
UGT1A6	UDP glucuronosyltransferase family 1-member A6	1.24	1.35
VEPH1	ventricular zone expressed PH domain containing 1	0.97	1.63

Table S3. mRNA downregulated in PCSK9-siRNA vs scramble-siRNA.

Symbol	Gene Name	Log (FC)	Log10 (P adjusted)
ATP8B3	ATPase phospholipid transporting 8B3	-0.64	1.51
CCDC113	coiled-coil domain containing 113	-0.94	2.05
CCDC183	coiled-coil domain containing 183	-0.96	3.39
CDH15	cadherin 15	-0.89	1.59
CDKN2C	cyclin dependent kinase inhibitor 2C	-0.74	2.56
CRIP1	cysteine rich protein 1	-0.78	4.14
CRYL1	crystallin lambda 1	-0.65	2.75
DHODH	dihydroorotate dehydrogenase (quinone)	-0.80	9.79
ENTPD2	ectonucleoside triphosphate diphosphohydrolase 2	-1.13	1.48
FLNC	filamin C	-0.71	13.67
HSD11B2	hydroxysteroid 11-beta dehydrogenase 2	-0.62	6.84
ID1	inhibitor of DNA binding 1, HLH protein	-0.91	8.96
INHBB	inhibin subunit beta B	-1.15	2.80
IRF8	interferon regulatory factor 8	-0.75	4.96
ITGA2	integrin subunit alpha 2	-0.63	2.09
KCNAB2	potassium voltage-gated channel subfamily A regulatory beta subunit 2	-0.62	1.72
KCNIP2	potassium voltage-gated channel interacting protein 2	-1.05	1.47
LBP	lipopolysaccharide binding protein	-1.15	1.67
MEIS3	Meis homeobox 3	-1.03	2.89
MTURN	maturin, neural progenitor differentiation regulator homolog	-0.61	1.57
NOTCH2NLB	notch 2 N-terminal like B	-0.79	1.38
NRM	Nurim	-0.69	4.62
PAQR4	progestin and adipoQ receptor family member 4	-0.77	4.45
PCDHGB6	protocadherin gamma subfamily B, 6	-0.78	1.93
PCSK9	proprotein convertase subtilisin/kexin type 9	-0.90	15.69
PCYOX1L	prenylcysteine oxidase 1 like	-0.64	1.78
PIK3IP1	phosphoinositide-3-kinase interacting protein 1	-0.69	2.17
PPDPF	pancreatic progenitor cell differentiation and proliferation factor	-0.71	13.70
PRR15L	proline rich 15 like	-0.90	7.17
PTH1R	parathyroid hormone 1 receptor	-1.41	1.82
PTPRU	protein tyrosine phosphatase receptor type U	-0.71	3.02
RIMBP3B	RIMS binding protein 3B	-0.84	1.66
RIPOR2	RHO family interacting cell polarization regulator 2	-1.83	2.12
RPUSD4	RNA pseudouridine synthase D4	-1.04	25.60
SHKBP1	SH3KBP1 binding protein 1	-0.63	12.87
SLC16A6	solute carrier family 16 member 6	-0.89	1.83
SLC43A2	Solute Carrier Family 43 Member 2	-0.71	2.51
SPINT1	serine peptidase inhibitor, Kunitz type 1	-0.71	2.12
TLE6	TLE family member 6, subcortical maternal complex member	-0.73	3.02
TMEM141	transmembrane protein 141	-0.60	6.35
TMEM150C	transmembrane protein 150C	-0.67	1.63

TMPRSS6	transmembrane serine protease 6	-0.67	1.46
TNS2	tensin 2	-0.68	2.77
TRIM29	tripartite motif containing 29	-0.90	1.66

Table S4. mRNA upregulated in PCSK9-siRNA vs scramble-siRNA.

**Symbol**	**Gene Name**	**Log (FC)**	**Log10 (P adjusted)**
A2M	alpha-2-macroglobulin	0.61	6.64
ADAM23	ADAM metallopeptidase domain 23	0.75	5.90
AKR1C1	aldo-keto reductase family 1 member C1	1.00	26.83
AKR1C2	aldo-keto reductase family 1 member C2	1.12	49.12
APOD	apolipoprotein D	0.93	1.85
CCN1	cellular communication network factor 1	0.76	3.96
CEACAM1	CEA cell adhesion molecule 1	0.89	17.54
CES1	NA	0.88	13.37
CLIP4	CAP-Gly domain containing linker protein family member 4	0.61	3.71
CNTNAP2	NA	0.72	3.87
CYP1A1	cytochrome P450 family 1 subfamily A member 1	1.06	21.25
DCDC1	doublecortin domain containing 1	0.89	7.27
DIO1	iodothyronine deiodinase 1	1.13	1.34
EDN1	endothelin 1	0.63	1.41
EGR1	early growth response 1	0.60	1.41
EMP3	epithelial membrane protein 3	0.66	1.48
F2RL2	coagulation factor II thrombin receptor like 2	1.64	5.16
FUT3	fucosyltransferase 3 (Lewis blood group)	1.49	2.89
G0S2	G0/G1 switch 2	0.90	5.30
GPR146	G protein-coupled receptor 146	0.61	1.44
GPRC5B	G protein-coupled receptor class C group 5 member B	0.63	2.71
GPX2	glutathione peroxidase 2	0.84	5.91
H4C8	H4 clustered histone 8	0.68	2.71
HHIP	hedgehog interacting protein	1.12	1.41
HKDC1	hexokinase domain containing 1	0.71	2.89
HTR3B	5-hydroxytryptamine receptor 3B	1.29	2.65
INSIG1	insulin induced gene 1	0.81	8.96
LRRC7	leucine rich repeat containing 7	0.63	1.90
MBOAT4	membrane bound O-acyltransferase domain containing 4	0.84	2.89
MEDAG	mesenteric estrogen dependent adipogenesis	1.12	1.79
MEGF10	multiple EGF like domains 10	0.70	1.65
NABP1	nucleic acid binding protein 1	0.63	3.74
NCF2	neutrophil cytosolic factor 2	1.17	1.53
NRG1	neuregulin 1	1.06	4.61
OAS1	2'-5'-oligoadenylate synthetase 1	0.64	4.61
OSGIN1	oxidative stress induced growth inhibitor 1	0.61	6.92
PCDH7	protocadherin 7	0.97	2.11
PCED1B	PC-esterase domain containing 1B	0.60	3.40
PDE5A	phosphodiesterase 5A	0.71	3.93
PGC	progastricsin	1.08	6.35
PHLDA1	pleckstrin homology like domain family A member 1	0.61	5.64
PLA1A	phospholipase A1 member A	0.65	5.15

PLCXD3	phosphatidylinositol specific phospholipase C X domain containing 3	1.22	1.37
PON1	paraoxonase 1	0.62	3.89
PRSS23	serine protease 23	0.87	5.35
PTGR1	prostaglandin reductase 1	0.97	27.24
QPCT	glutaminyl-peptide cyclotransferase	0.64	2.12
RAB17	RAB17, member RAS oncogene family	0.85	2.23
ROS1	ROS proto-oncogene 1, receptor tyrosine kinase	0.62	2.56
SLC1A3	solute carrier family 1 member 3	0.90	5.25
SLC5A3	solute carrier family 5 member 3	0.63	6.39
SLC7A7	solute carrier family 7 member 7	0.67	3.41
SLCO1B3	solute carrier organic anion transporter family member 1B3	0.62	2.72
SPTSSB	serine palmitoyltransferase small subunit B	0.64	1.95
SSUH2	ssu-2 homolog	1.33	7.50
TENM1	teneurin transmembrane protein 1	0.65	1.68
TENT5C	terminal nucleotidyltransferase 5C	1.12	4.51

Table S5. mRNA downregulated in ANGPTL3/PCSK9-siRNA vs scramble-siRNA.

Symbol	Gene Name	Log (FC)	Log10 (P adjusted)
ACKR3	atypical chemokine receptor 3	-1.06	1.76
ADAMTS 10	ADAM metallopeptidase with thrombospondin type 1 motif 10	-0.85	1.96
ADAP1	ArfGAP with dual PH domains 1	-1.02	2.59
AHSG	alpha 2-HS glycoprotein	-0.67	4.62
AIFM3	apoptosis inducing factor mitochondria associated 3	-0.81	1.64
ANGPTL3	angiopoietin like 3	-2.56	115.2
ARRDC3	arrestin domain containing 3	-0.62	7.94
ATP6V1G 1	ATPase H+ transporting V1 subunit G1	-0.61	10.92
B4GALNT 4	beta-1,4-N-acetyl-galactosaminyltransferase 4	-0.75	1.96
B4GALT5	beta-1,4-galactosyltransferase 5	-1.11	36.53
BGN	biglycan	-0.65	2.04
BHLHA15	basic helix-loop-helix family member a15	-0.65	8.72
BSN	bassoon presynaptic cytomatrix protein	-0.65	3.45
C18orf32	chromosome 18 open reading frame 32	-0.75	4.3
C5orf24	chromosome 5 open reading frame 24	-0.76	18.57
C9orf40	chromosome 9 open reading frame 40	-0.6	2.56
CBFB	core-binding factor subunit beta	-0.66	12.12
CCDC183	coiled-coil domain containing 183	-1.27	6.4
CCNJ	cyclin J	-0.66	11.34
CD14	CD14 molecule	-0.59	1.8
CDH15	cadherin 15	-0.75	1.35
CLCN2	chloride voltage-gated channel 2	-0.62	3.12
CLIC6	chloride intracellular channel 6	-0.67	3.28
COL9A3	collagen type IX alpha 3 chain	-0.6	1.51
COX14	cytochrome c oxidase assembly factor COX14	-0.69	8.49
CRIP1	cysteine rich protein 1	-0.91	6.09
CYS1	cystin 1	-0.96	1.52
DDAH1	dimethylarginine dimethylaminohydrolase 1	-0.68	10.39
DENND6 A	DENN domain containing 6A	-0.76	11.05
DHODH	dihydroorotate dehydrogenase (quinone)	-0.76	9.06
DLX6	distal-less homeobox 6	-0.67	4.93
DPY19L1	dpy-19 like C-mannosyltransferase 1	-0.71	13.81
DYNLL2	dynein light chain LC8-type 2	-0.61	14.85
EIF5A2	eukaryotic translation initiation factor 5A2	-0.83	6.94
ELK3	ETS transcription factor ELK3	-0.82	11.07
ENPP2	ectonucleotide pyrophosphatase/phosphodiesterase 2	-0.6	15.07
ESPN	espin	-0.62	5.8
F2RL1	F2R like trypsin receptor 1	-0.65	2.9
FBXL20	F-box and leucine rich repeat protein 20	-0.74	13.96
FBXO2	F-box protein 2	-0.66	1.35

FBXO21	F-box protein 21	-0.61	11.28
FBXO32	F-box protein 32	-0.81	3.55
FGD1	FYVE, RhoGEF and PH domain containing 1	-0.71	2.61
FLNC	filamin C	-0.64	11.41
FNDC10	fibronectin type III domain containing 10	-0.84	1.42
FZD2	frizzled class receptor 2	-0.62	2.27
GIPC2	GIPC PDZ domain containing family member 2	-1.21	9.55
GLUD1	glutamate dehydrogenase 1	-0.89	33.06
GPR153	G protein-coupled receptor 153	-0.94	1.66
HAVCR1	hepatitis A virus cellular receptor 1	-0.79	15.9
HCN2	hyperpolarization activated cyclic nucleotide gated potassium and sodium channel 2	-0.6	1.96
HECA	hdc homolog, cell cycle regulator	-0.92	17.76
HELB	DNA helicase B	-1.02	3.83
HFE	homeostatic iron regulator	-0.77	3.39
HNF1B	HNF1 homeobox B	-0.63	9.5
HSD11B2	HNF1 homeobox B	-0.84	13.36
HTRA3	hydroxysteroid 11-beta dehydrogenase 2	-0.68	1.51
IFITM2	HtrA serine peptidase 3	-0.77	1.59
IGFBP6	interferon induced transmembrane protein 2	-0.7	2.38
IL17RE	insulin like growth factor binding protein 6	-1.11	2.32
IL1RAP	interleukin 17 receptor E	-0.74	11.15
INO80B	interleukin 1 receptor accessory protein	-0.61	2.68
INPP5A	INO80 complex subunit B	-0.65	10.53
IQCA1	inositol polyphosphate-5-phosphatase A	-1.06	1.59
IRF8	IQ motif containing with AAA domain 1	-0.91	8.1
KCNJ4	interferon regulatory factor 8	-0.87	1.35
KIF12	kinesin family member 12	-0.66	4.95
KLHL26	kelch like family member 26	-1.41	2.61
LDAH	lipid droplet associated hydrolase	-0.77	9.95
LEFTY1	left-right determination factor 1	-0.78	1.51
LGI4	leucine rich repeat LGI family member 4	-2.19	2.18
LMAN1	lectin, mannose binding 1	-1.02	27.61
MEIS3	Meis homeobox 3	-0.69	1.5
MELTF	melanotransferrin	-0.64	2.42
MISP3	MISP family member 3	-0.59	1.59
MOCS2	molybdenum cofactor synthesis 2	-0.78	6.82
MPV17L	MPV17 mitochondrial inner membrane protein like	-0.64	8.96
MTURN	maturin, neural progenitor differentiation regulator homolog	-0.95	4.4
NDNF	neuron derived neurotrophic factor	-0.69	1.38
NKAIN1	sodium/potassium transporting ATPase interacting 1	-0.66	1.84
NOTCH2 NLB	NA	-0.92	2.24
NPDC1	neural proliferation, differentiation and control 1	-0.69	2.67
NPEPL1	aminopeptidase like 1	-0.59	3.41

NPTX2	neuronal pentraxin 2	-0.75	6.09
NR0B2	nuclear receptor subfamily 0 group B member 2	-0.65	5.29
P2RX4	purinergic receptor P2X 4	-0.68	9.09
PAFAH1B2	platelet activating factor acetylhydrolase 1b catalytic subunit 2	-0.62	12.76
PAQR4	progestin and adipoQ receptor family member 4	-0.75	4.48
PCSK9	proprotein convertase subtilisin/kexin type 9	-1.04	21.63
PIGY	phosphatidylinositol glycan anchor biosynthesis class Y	-1.01	15.82
PIK3IP1	phosphoinositide-3-kinase interacting protein 1	-0.7	2.58
PNCK	pregnancy up-regulated nonubiquitous CaM kinase	-2.67	2.62
POM121	POM121 transmembrane nucleoporin	-0.8	26.94
POM121C	POM121 transmembrane nucleoporin C	-0.83	24.95
PRR15L	proline rich 15 like	-0.96	8.61
PRXL2B	peroxiredoxin like 2B	-0.6	3.37
PTH1R	parathyroid hormone 1 receptor	-1.28	1.77
PTPRJ	protein tyrosine phosphatase receptor type J	-0.59	13.19
PTPRU	protein tyrosine phosphatase receptor type U	-0.66	2.9
PYURF	PIGY upstream open reading frame	-1.12	16.84
RAB2A	RAB2A, member RAS oncogene family	-0.85	22.36
RAB40B	RAB40B, member RAS oncogene family	-0.88	4.58
RASSF5	Ras association domain family member 5	-0.91	12.32
RPUSD4	RNA pseudouridine synthase D4	-0.97	22.15
SERPINA1	serpin family A member 1	-0.9	13.76
SERPINA6	serpin family A member 6	-0.69	5.26
SHKBP1	SH3KBP1 binding protein 1	-0.62	12.32
SLC16A6	solute carrier family 16 member 6	-0.74	1.51
SLC22A5	solute carrier family 22 member 5	-0.73	4.3
SLC38A6	solute carrier family 38 member 6	-0.61	4.69
SLC9A7	solute carrier family 9 member A7	-0.96	8.17
SMC1A	structural maintenance of chromosomes 1A	-0.77	24.99
SNAPIN	SNAP associated protein	-0.61	8.38
SPINT1	serine peptidase inhibitor, Kunitz type 1	-0.59	1.71
STMN3	stathmin 3	-0.59	3.16
TIMP2	TIMP metallopeptidase inhibitor 2	-0.62	5.34
TMEM121	transmembrane protein 121	-2.07	2.22
TMEM150C	transmembrane protein 150C	-0.73	2.25
TMEM200B	transmembrane protein 200B	-0.64	4.85
TMEM217	transmembrane protein 217	-1.16	1.39
TMEM30B	transmembrane protein 30B	-0.73	1.44
TMEM64	transmembrane protein 64	-0.68	6.96
TMEM69	transmembrane protein 69	-0.6	9.62

TMPRSS6	transmembrane serine protease 6	-0.72	2
TMTC2	transmembrane O-mannosyltransferase targeting cadherins 2	-1.16	3.47
TNS2	tensin 2	-0.69	3.2
TPRG1L	tumor protein p63 regulated 1 like	-0.96	21.55
TRIM29	tripartite motif containing 29	-0.99	2.29
TRPM2	transient receptor potential cation channel subfamily M member 2	-0.64	2.15
TTC8	tetratricopeptide repeat domain 8	-0.99	6.62
UCK2	uridine-cytidine kinase 2	-0.74	19.41
UCP2	uncoupling protein 2	-0.66	5.19
UHRF1	ubiquitin like with PHD and ring finger domains 1	-0.64	3.67
UXS1	UDP-glucuronate decarboxylase 1	-0.61	9.19
VWA1	von Willebrand factor A domain containing 1	-0.64	4.95
WNT5B	Wnt family member 5B	-0.63	1.75
ZNF100	zinc finger protein 100	-0.66	3.48

Table S6. mRNA upregulated in ANGPTL3/PCSK9-siRNA vs scramble-siRNA.

Symbol	Gene Name	Log (FC)	Log10 (P adjusted)
ACSL5	acyl-CoA synthetase long chain family member 5	0.68	9.96
ADAM23	ADAM metallopeptidase domain 23	0.77	6.8
AKAP12	A-kinase anchoring protein 12	0.61	5.5
ALPK3	alpha kinase 3	0.6	3.6
ANKRD1	ankyrin repeat domain 1	1.28	4.16
ANKS1B	ankyrin repeat and sterile alpha motif domain containing 1B	0.61	5.29
ANXA1	annexin A1	0.71	2.12
APOD	apolipoprotein D	1.35	4.95
ARHGAP 19	Rho GTPase activating protein 19	0.63	4.62
ATP10B	ATPase phospholipid transporting 10B (putative)	0.96	1.72
BATF3	basic leucine zipper ATF-like transcription factor 3	1.28	1.34
CCDC65	coiled-coil domain containing 65	0.89	1.33
CCN1	cellular communication network factor 1	1.02	8.32
CD36	CD36 molecule	1.53	2.09
CEACAM 1	CEA cell adhesion molecule 1	0.64	8.62
CES1	carboxylesterase 1	0.95	16.29
CFAP251	cilia and flagella associated protein 251	0.7	1.36
CFAP91	cilia and flagella associated protein 91	1.36	1.38
CNTNAP 2	contactin associated protein 2	1.08	10.58
CXCL6	C-X-C motif chemokine ligand 6	0.77	1.6
CYP2B6	cytochrome P450 family 2 subfamily B member 6	0.62	6.65
DCBLD1	discoidin, CUB and LCCL domain containing 1	0.69	9.25
DDX60	DExD/H-box helicase 60	0.69	2.71
DIO2	iodothyronine deiodinase 2	1.6	1.68
DLG2	discs large MAGUK scaffold protein 2	1.18	1.99
DNAH2	dynein axonemal heavy chain 2	1.42	1.88
DNAH5	dynein axonemal heavy chain 5	0.83	1.61
DSG3	desmoglein 3	0.62	4.06
EDN1	endothelin 1	0.92	3.85
EPHA6	EPH receptor A6	0.68	3.55
ESRRG	estrogen related receptor gamma	0.72	1.76
FAT2	FAT atypical cadherin 2	1.14	1.99
G0S2	G0/G1 switch 2	1.08	8.69
GABRG1	gamma-aminobutyric acid type A receptor subunit gamma1	0.83	5.91
GASK1B	golgi associated kinase 1B	0.81	7.72
GRM3	glutamate metabotropic receptor 3	0.69	2.01
HDAC9	histone deacetylase 9	1.03	8.47
HHIP	hedgehog interacting protein	1.1	1.64
HSD17B6	hydroxysteroid 17-beta dehydrogenase 6	0.98	2.04
HTR2C	5-hydroxytryptamine receptor 2C	1.57	2.01

IL15	interleukin 15	0.81	2.34
IQCN	IQ motif containing N	0.72	1.34
IRF6	interferon regulatory factor 6	1.79	4.58
IRF9	interferon regulatory factor 9	0.64	8.2
KLKB1	kallikrein B1	0.93	1.72
KRT23	keratin 23	0.64	1.45
LRRC7	leucine rich repeat containing 7	0.69	2.7
MEDAG	mesenteric estrogen dependent adipogenesis	1.3	2.9
MGAM	maltase-glucoamylase	0.8	11.34
MLIP	muscular LMNA interacting protein	1.52	2.73
MX1	MX dynamin like GTPase 1	0.85	2.51
MYOF	myoferlin	0.82	3.44
NABP1	nucleic acid binding protein 1	0.75	6.04
NOSTRIN	nitric oxide synthase trafficking	0.61	7.18
OAS1	2'-5'-oligoadenylate synthetase 1	0.93	11.7
OAS3	2'-5'-oligoadenylate synthetase 3	0.78	17.99
PAG1	phosphoprotein membrane anchor with glycosphingolipid microdomains 1	0.65	7.42
PCED1B	PC-esterase domain containing 1B	0.82	7.63
PHLDA1	pleckstrin homology like domain family A member 1	0.76	10.15
PLA2G4A	phospholipase A2 group IVA	0.63	2.2
PLCXD3	phosphatidylinositol specific phospholipase C X domain containing 3	1.42	2.31
POLR3G	RNA polymerase III subunit G	0.69	2.04
PRSS23	serine protease 23	1.16	11.03
QPCT	glutaminyl-peptide cyclotransferase	0.84	4.45
RAB17	RAB17, member RAS oncogene family	0.73	1.85
RASGEF1B	RasGEF domain family member 1B	1.06	2.68
RNASE1	ribonuclease A family member 1, pancreatic	2.22	2.7
RUNX1	RUNX family transcription factor 1	0.6	4.3
SAMD9L	sterile alpha motif domain containing 9 like	2.25	1.32
SLC10A4	solute carrier family 10 member 4	0.76	1.58
SLC1A3	solute carrier family 1 member 3	1.04	7.99
SPON1	spondin 1	1.64	2.22
TENM1	teneurin transmembrane protein 1	0.74	2.59
TENT5C	terminal nucleotidyltransferase 5C	1.2	5.72
THBS1	thrombospondin 1	0.6	7.57
TLR4	toll like receptor 4	1.57	1.54
TMC7	transmembrane channel like 7	0.66	9.6
TNFRSF19	TNF receptor superfamily member 19	0.94	17.26
UGT1A3	UDP glucuronosyltransferase family 1 member A3	1.46	1.98
UGT1A6	UDP glucuronosyltransferase family 1 member A6	1.95	3.77
VCAN	versican	0.75	21.77
ZNF789	zinc finger protein 789	0.59	2.2
